# Is Vestibular Neuropathy Rather a Neuritis?

**DOI:** 10.7759/cureus.29959

**Published:** 2022-10-05

**Authors:** Sophia M Haeussler, Samira I Zabaneh, Miriam Stegemann, Heidi Olze, Arne Böttcher, Katharina Stölzel

**Affiliations:** 1 Otolaryngology - Head and Neck Surgery, UKE Hamburg, Hamburg, DEU; 2 Otolaryngology - Head and Neck Surgery, Charité Berlin, Berlin, DEU; 3 Infectious Disease, Charité Berlin, Berlin, DEU; 4 Otolaryngology, Charité Berlin, Berlin, DEU

**Keywords:** vestibular dysfunction, vertigo diagnosis, head impulse test, herpes simplex, lyme's disease, vestibular neuritis

## Abstract

Background/objectives

Vestibular neuritis (VN) is one of the most common causes of peripheral vestibular neuropathy. The goal of this study is to investigate the possible infectious causes of VN in a large cohort of patients.

Material and methods

In total, 98 consecutive VN patients were enrolled in this retrospective study over a four-year period (04/2015-04/2019). Diagnosis of VN was made by clinical examination and functional diagnostics. We focused on infectious causes such as neurotropic viruses and Lyme disease (LD) and evaluated infection parameters as well as the concomitant diseases.

Results

In this cohort, we found pathologically elevated leukocytes or C-reactive protein (CRP) levels, and/or acute herpes simplex virus (HSV) infection, cytomegalovirus (CMV) infection and LD in 42 patients (42.85%). Leukocytes were elevated in 39 of 98 patients (39.8%) and the mean count was 9717 ± 2991 /μl. The group comparison between patients with vestibular loss (n=42) and patients with vestibular hypofunction (n=45) revealed a significant difference in regard to elevated leukocytes (p=0.028). In total, 28 of 53 patients (52.8%) were positive for HSV immunoglobulin (Ig) G and four of 53 patients were positive for HSV IgM (7.5%). Six of 53 patients (11.3%) were positive for LD IgM.

Conclusion

In this cohort, there was a large number of VN patients with infectious signs; several patients tested positive for HSV and LD. Therefore we recommend testing VN patients not only for HSV but also for LD and other neurotropic viruses. This approach enables to complement the standard VN treatment with a specific treatment.

## Introduction

Vestibular neuritis (VN) is one of the most common causes of peripheral vestibular neuropathy (PVP) and has an incidence of 3.5-15/100 000 [1-3]. Common synonyms are vestibular neuronitis or neuropathy, acute unilateral vestibular paralysis [4, 5], acute unilateral peripheral vestibulopathy and epidemic vertigo [6,7]. There is an equal distribution between male and female patients and the age of onset is between 30 and 60 years [4,7,8].

Dix and Hallpike (1949/1952) [6]were the first to introduce the term vestibular neuronitis and, in their study, they explained the typical symptoms of VN. They reported symptoms of rotational vertigo and disequilibrium, which were aggravated by head movements, and they noted that patients had normal cochlear function. They also found a high prevalence of febrile or ear, nose and throat infections in VN patients.

VN is characterized by acute rotational vertigo or dizziness, together with nausea and vomiting, horizontal spontaneous nystagmus, and gait and postural instability with a tendency to fall to the affected side. The onset is acute (minutes to hours) and lasts up to six weeks [7-9]. The disease is caused by a loss of vestibular function either by a lesion of the vestibular nerve or the vestibular labyrinth. Some earlier studies have concluded that most cases of VN are due to a lesion of the superior vestibular nerve or its innervated organs, which are the anterior and horizontal semicircular canal and the utricle. In rare cases, the inferior vestibular nerve, which innervates the posterior semicircular canal and the saccule, is thought to be affected [10-13]. There have also been studies on the anatomic differences between the superior, inferior and singular vestibular nerve and there is histopathologic evidence that, in patients with VN, there is a degeneration of vestibular nerve fibers, especially of the superior vestibular nerve [14-16].

Another theory states that VN is caused by viral infections or reactivation of latent infections with viruses such as herpes simplex virus type 1 (HSV-1) and type 6 (HSV-6) [17-20]. The infection or reactivation may cause damage to the ganglion cells and axons of the vestibular nerve. Another related theory is that VN is caused by inflammatory and immunological mechanisms in patients with elevated levels of C-reactive protein (CRP) and elevated peripheral blood mononuclear cells (PBMCs), which may lead to aggregates and consecutively to thrombotic events and hypoperfusion of the nerve [21,22]. Chung et al. [14] were the first to evaluate the nonspecific infection parameters neutrophil to lymphocyte ratio (NLR) and platelet to lymphocyte ratio (PLR) for VN patients. They found elevated ratios in VN patients compared to healthy individuals.

The aim of the study is the evaluation of inflammation as a cause of VN with clinical and laboratory findings like leukocytes, CRP levels as well as NLR and PLR and to evaluate the impact of concomitant diseases including cardiovascular diseases and specific inflammatory diseases on VN.

## Materials and methods

Following institutional review board approval (application no.: EA1/181/19), we performed an International Classification of Diseases (ICD)-10-based study on vestibular neuritis (H81.2-; H81.8-; H81.9-) at a tertiary referral center, using the SAP database system (SAP Deutschland SE & Co. KG, Walldorf, Germany) containing patients’ records over a four-year period (04/2015-04/2019) to identify clinically and laboratory-confirmed cases of VN in adult patients. Written informed consent was obtained from each patient included in this study.

The inclusion criteria are: acute onset of severe rotational vertigo, which lasts for at least 24 hours and up to 6 weeks, spontaneous peripheral vestibular nystagmus (horizontal-torsional direction-fixed, trajectory appropriate to the involved side), the age of patients >18 years, a positive caloric testing and/or positive clinical Head Impulse Test (HIT) with horizontal saccades and the symptoms are not better accounted for another disorder. The exclusion criteria are: cochlear symptoms (deafness, sudden hearing loss, tinnitus), additional neurological symptoms, other pathological findings except from cranial nerve eight (CNVIII) affection of the or enlarged vestibular aqueduct or inner ear canal abnormities, Menière’s disease and benign paroxysmal positional vertigo (BPPV), negative caloric testing and negative clinical HIT and post-concussion diagnosis.

The patients with VN were admitted to the hospital as medical emergencies and were diagnosed by ear, nose and throat (ENT) and neurology specialists. In cases of suspected vestibular pathology, the diagnostics were performed according to specific guidelines at the tertiary referral center. They included a focused anamnesis, clinical examination, and especially in cases with a positive anamnesis a serologic blood test for neurotropic infectious diseases such as herpes simplex virus (HSV), cytomegalovirus (CMV), Epstein-Barr virus (EBV), varicella zoster virus (VZV), *Borrelia burgdorferi*, toxoplasmosis, which were tested with Enzyme-Linked Immunosorbent Assay and *Treponema pallidum*, which was tested with microimmunoassay. The serologic blood test for *Borrelia burgdorferi *followed a two-step algorithm. When in the first step IgG and IgM antibodies were detected, then in the second step antigens recognized by the antibodies were determined by using separate IgG and IgM immunoblots. Laboratory testing included a differential blood count in some cases, so the neutrophil-lymphocyte ratio (NLR) and the platelet-lymphocyte ratio (PLR) were calculated for these patients. Vestibular testing was performed with clinical examination, caloric testing, and in some cases with Video HIT (vHIT). As a consequence of the lack of availability of vHIT and vestibular evoked myogenic potentials (VEMP) during the study period, vHIT was performed only in some cases.

If there were uncertain findings in the neurological clinical examination, cranial magnetic resonance imaging (cMRI) and/or computed tomography (CT) angiography of the head and neck was performed to rule out the possibility of a stroke or intracranial bleeding. cMRI with constructive interference in steady state (CISS) and MP-RAGE/VIBE post-contrast sequences was performed to confirm peripheral vestibular disorder, and the stroke protocol was used (T2/turbo inversion recovery magnitude (TIRM), diffusion-weighted imaging (DWI), susceptibility-weighted imaging (SWI)) to examine the symptom of central dizziness.

The data for this study was extracted from the SAP database system. It contained patient data, VN-focused anamnesis, including concomitant diseases and laboratory findings, including infection parameters (CRP, leukocytes, lymphocytes, neutrophils, platelets and serology for viral infections as described earlier) as well as the results of clinical examination with HIT. Additionally, data on vestibular testing and radiological findings were collected and evaluated. To decide whether the caloric test was pathological, we used the Jongkees formula [15]. The cut-off for the speed of the slow phase of the nystagmus was 5 percent. The results of caloric testing were divided into subgroups: non-excitability (no contralateral nystagmus), high-grade hypoexcitability (2-3 contralateral nystagmuses), hypoexcitability (side difference: > 25%) and low-grade hypoexcitability (side difference: < 25%). In 13 cases the caloric test could not be carried out. In these cases, the clinical examinations in the emergency room with spontaneous nystagmus towards the normal side using the Frenzel goggles, tendency to fall to the affected side and visible saccades when performing the HIT towards the affected side were used. All symptoms had to be consistent. 

According to our guidelines, the patients were treated with a prednisone tapering scheme, dimenhydrinate and vestibular rehabilitation with vestibular training as well as balance exercises. The patients were advised to perform vestibular rehabilitation exercises for central vestibular compensation also after discharge from the hospital and in cases of persistent VN symptoms. 

The group comparison was conducted using the Mann-Whitney U-Test and the significance was set at p < 0.05. Statistical analysis was performed using SPSS software (IBM SPSS Statistics for Windows, Version 24.0, IBM Corp., Armonk, USA).

## Results

Patient baseline characteristics

Patients’ baseline characteristics (number, age at diagnosis, sex, affected side, recurrence rate) were obtained by screening patients’ records, and are summarized in Table 1. In total, 98 patients were identified matching the inclusion criteria of this study, of which 56 (57.1%) were male and 42 (42.9%) were female patients. The mean age at diagnosis of VN was 50.3 ± 15.7 years (range: 18-86 years) (Table 1). Fifty-three (54.1%) suffered from VN of the left side, and 45 (45.9%) suffered from VN of the right side. Thirteen patients (13.3%) reported a recurrent event of sudden prolonged rotational vertigo; anamnestically they had been diagnosed with VN before and the symptoms did not fulfill the criteria for Menière's disease.

**Table 1:** General patient characteristics SD, standard deviation.

Variable	Value
Age, mean ± SD (range), years	50.3 ± 15.7 (18–86)
Number of patients, n	98
Sex, n (%)	
Male	56 (57.1)
Female	42 (42.9)
Affected side, n (%)	
Right	44 (44.4)
Left	50 (50.5)
Bilateral	4 (4.0)
Primary event, n (%)	85 (86.7)
Recurrence, n (%)	13 (13.3)

Clinical examination and functional diagnostics

On clinical examination, a horizontal spontaneous nystagmus was seen in 96 patients (98%), which was suppressed by visual fixation. A vestibulo-ocular reflex (VOR) deficit, tested with the Halmagyi head impulse test, was seen in 44 patients (44.9%). Functional diagnostics were performed one to five days after the onset of symptoms, and the results are summarized in Table 2. 

**Table 2:** Clinical findings and functional test results HIT, head impulse test; CRP, C-reactive protein; IgM, immunoglobulin M; HSV, herpes simplex virus; LD, Lyme disease; CMV, cytomegalovirus

Variable	Value
Spontaneous nystagmus, n (%)	
Total	98 (100)
Positive	96 (98)
Negative	2 (2)
Clinical HIT, n (%)	
Total	98 (100)
Positive	44 (44.9)
Negative	54 (55.1)
Caloric test, n (%)	
Total	77 (78.6)
Positive	75 (76.5)
Negative	2 (2.0)
Vestibular dysfunction in caloric test, n (%)	
Total	75 (78.6)
Vestibular non-excitability	41 (41.8)
Vestibular high-grade hypoexcitability	10 (10.4)
Vestibular hypoexcitability	22 (22.4)
Vestibular low-grade hypoexcitability	2 (2.0)
Elevated leukocytes	
Total, n (%)	39 (39.8)
Mean ± standard deviation	12 344 ± 1703 /μl
Minimum; Maximum	10 520 /μl; 16 910 /μl
Elevated CRP	
Total, n (%)	8 (8.2)
Mean ± standard deviation	21.9 ± 10.1 mg/dl
Minimum; Maximum	7.5 mg/dl; 38.9 mg/dl
Acute inflammation signs, n (%)	
Total	42 (42.9)
Acute infections with elevated IgM, n (%)	
Total	11 (20.8)
HSV	4 (7.5)
LD IgM positive LD IgG positive IgM + Immunoblot positive (Immunoblot)	6 (11.3) 3 (5.7) 2 (3.8)
CMV	1 (1.9)

Caloric testing was performed in 77 patients (78.6%). A vestibular dysfunction was confirmed by functional diagnostics in 75 patients (76.5%). Caloric testing revealed a vestibular non-excitability in 41 patients (41.8%), high-grade hypoexcitability (2-3 contralateral nystagmuses) in 10 patients (10.4%), hypoexcitability (side difference: > 25%) in 22 patients (22.4%) and low-grade hypoexcitability (side difference: < 25%) in two patients (2.0%). For the other patients, the bedside head impulse test (bHIT) was positive in 19 patients (19.4%) and negative in two patients (2.0%).

Laboratory findings

The mean of the leukocyte count (n=95) was 9717 ± 2991 /μl (minimum 3860/μl; maximum 16910/μl). The laboratory test results revealed elevated leukocytes in 39 of 95 patients (41.1%) with a mean value of 12 344 ± 1703 /μl (minimum 10520/μl; maximum 16910/μl); the cut-off score was 10500/μl. C-reactive protein (CRP) was elevated in eight of 98 patients (8.2%) with a mean value of 21.9 ± 10.1 mg/dl (Table 2). We found an acute infection with pathologically elevated leukocytes and/or C-reactive protein (CRP) levels, and/or positive Immunoglobulin M (IgM) levels for acute Lyme disease (LD, neuroborreliosis was ruled out by neurologic examination), herpes simplex (HSV) or cytomegalovirus (CMV) in 42 of 98 patients (42.9%).

**Figure 1:**
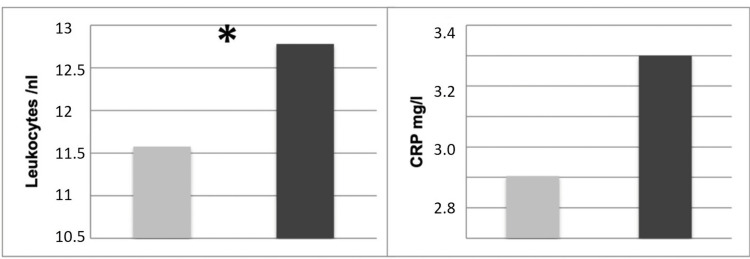
Group comparison between patients with vestibular loss (black bar) and patients with vestibular hypofunction (grey bar). The asterisk (*) indicates statistical significance.

The neutrophil to lymphocyte ratio (NLR) was 3.63 ± 3.21 and the platelet to lymphocyte ratio (PLR) was 147.75 ± 67.47. The group comparison between patients with vestibular loss and patients with vestibular hypofunction revealed a significant difference in regard to elevated leukocytes (p=0.028) (Figure 1 and Table 3). There were no statistically significant differences with respect to neutrophils (p=0.315), lymphocytes (p=0.927), thrombocytes (p=0.412), CRP (p=0.802), NLR (p=0.730) or PLR (p=0.190). 

**Table 3:** Baseline characteristics and comparison of the group with vestibular loss and the group with vestibular hypofunction. NLR, neutrophil to lymphocyte ratio; PLR, platelet to lymphocyte ratio; CRP, C-reactive protein

	Neutrophiles /nl	Lymphocytes /nl	Platelets /nl	NLR	PLR	CRP mg/dl	Elevated leukocytes /nl
Vestibular loss	5.59 ± 2.72	2.24 ± 1.42	236.38 ± 66.66	3.63 ± 3.21	147.75 ± 67.74	3.3 ± 7.5	12.78 ± 1.99 (n=20)
Vestibular hypofunction	6.20 ± 3.57	1.67 ± 0.62	237.75 ± 42.29	4.91 ± 4.89	155.56 ± 47.72	2.9 ± 5.1	11.58 ± 0.69 (n=12)

The test results for viral infections (total n=53) revealed elevated HSV immunoglobulin G (IgG) in 28 patients (52.8%) and elevated HSV immunoglobulin M (IgM) in four patients (7.5%). CMV IgG was elevated in 24 patients (45.3%) and CMV IgM in one patient (1.9%). Serology provides indirect evidence of recent or prior CMV infection based upon changes in antibody titers at different time points during a clinical illness. Serologic tests are also helpful in determining past exposure to CMV infection. When assessing past CMV exposure or infection, any IgG result above the cutoff of the test is considered positive. There was positive IgG for EBV in 19 patients (35.8%) and for VZV in 40 patients (75.5%); the latter high prevalence is probably due to the VZV vaccination. There was no positive IgM for EBV or VZV. Six patients (11.32%) tested positive for LD IgM in the screening test, the immunoblot was positive in two patients (3.8%). None were positive for *Treponema pallidum *or toxoplasmosis.

Comorbidities

The most frequent underlying disease was arterial hypertension (n=17, 17.34%), followed by type 2 diabetes mellitus (n=7; 7.14%), upper airway infections (n=5; 5.10%), coronary heart disease, atrial fibrillation, psoriasis and depression (all n=2; 2.04%). See Table 4for the list of concomitant diseases present in our study population.

**Table 4:** Concomitant diseases of the study population (n=98).

Concomitant disease	n (%)
Arterial hypertension	17 (17.34)
Type 2 diabetes mellitus	7 (7.14)
Upper respiratory infection	5 (5.1)
Coronary heart disease	2 (2.04)
Psoriasis	2 (2.04)
Atrial fibrillation	2 (2.04)
Depression	2 (2.04)
Bronchial asthma	1 (1.02)
Atypical hemolytic uremic syndrome (aHUS)	1 (1.02)
Chronic urethritis	1 (1.02)
Thrombocytosis	1 (1.02)
HIV	1 (1.02)
Deep vein thrombosis	1 (1.02)
Hyperlipidemia	1 (1.02)
Migraine	1 (1.02)
Infectious tendency/pneumonia	1 (1.02)
Kidney transplantation	1 (1.02)
Abscesses (liver/lungs)	1 (1.02)

Imaging findings

**Figure 2:**
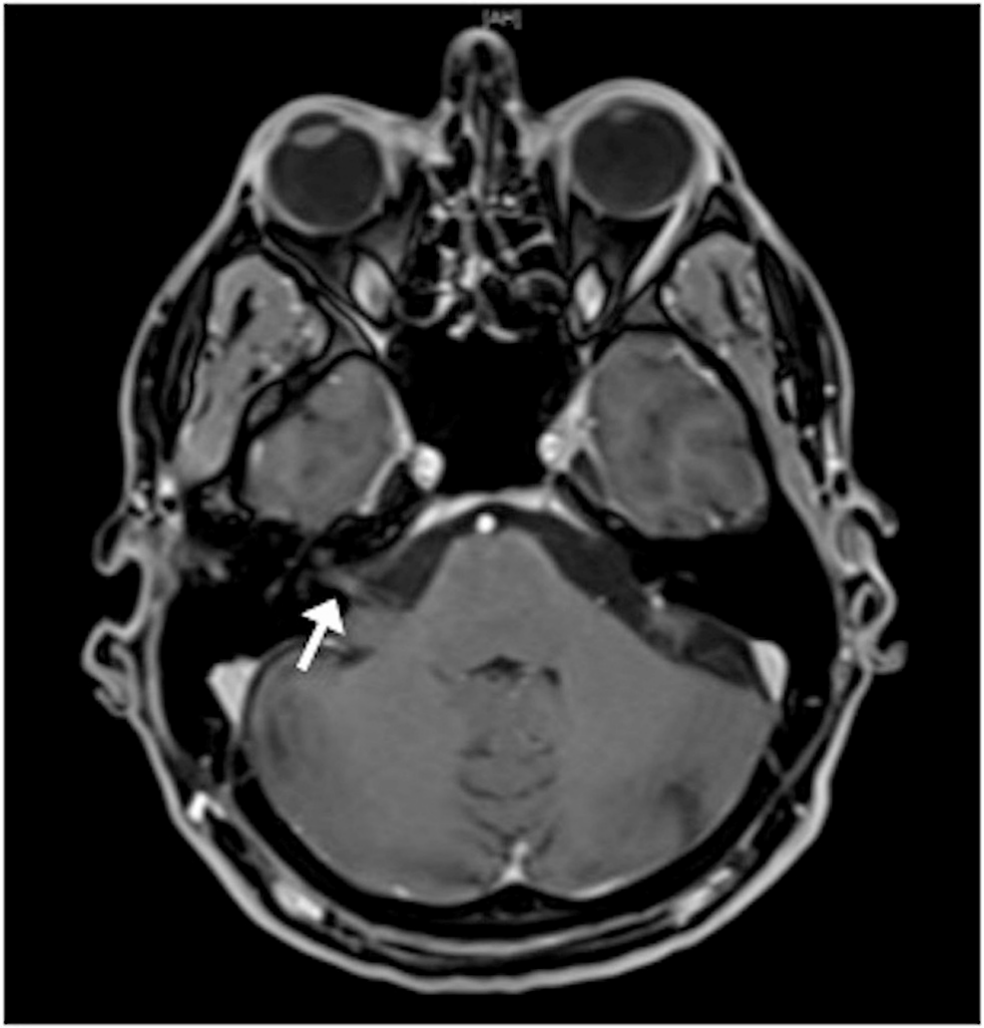
cMRI showing an enhancement of the right vestibulocochlear nerve. cMRI, cranial magnetic resonance imaging

In 30 of 98 patients (30.6%), a contrast-enhanced cMRI was performed to eliminate the possibility of a stroke, intracranial bleeding or structural abnormalities. In one patient, the MR images showed an enhancement of the vestibular nerve in the delayed post-contrast MRI sequences (Figure 2). In 17 of 98 patients (17.34%), CT angiography of the head was performed as emergency imaging.

## Discussion

Patient baseline characteristics

In this study, the gender distribution of VN did not differ significantly between men and women (56 male (57.1%); 42 female patients (42.9%)). In previous studies by Dix and Hallpike [6] and Sekitani et al. [3], there was also no difference in the gender distribution. In our study, the mean age at diagnosis was 50.3 years, which coincides with the results of earlier studies [3,5,6] with a typical age of onset between 30 and 60 years. The recurrence rate of VN in this study was 13.3%, which is higher compared to the study by Huppert et al. [16]. In their long-term follow-up study of 103 VN patients, 1.9% experienced a second VN on the contralateral side. In the review by Greco et al. [5], the authors stated that there was no recurrence of VN on the same side. The high recurrence rate in this study might be due to retrospective data without available functional diagnostics of the prior episodes of rotational vertigo.

Clinical examination and functional diagnostics

In the clinical examination, there was no spontaneous horizontal nystagmus seen in two of the 98 patients (2%). The clinical examination took place when the patients were admitted to the hospital, which in two cases was after the acute onset of the VN symptoms and therefore vestibular dysfunction was partly compensated. Consequently, horizontal spontaneous nystagmus was not seen in all patients. Functional diagnostics were performed one to five days after the onset of symptoms, and a vestibular dysfunction was confirmed by functional diagnostics in 75 patients (76.5%). Nonetheless, the other patients were also diagnosed with VN as they had the typical anamnesis and clinical signs (spontaneous horizontal nystagmus, positive HIT) when admitted to the hospital. A VOR deficit, tested with Halmagyi bedside HIT, was only seen in 44 patients (44.9%). According to Yip et al. [17] the bedside HIT has moderate sensitivity and low positive predictive value. So the authors conclude, that "the bHIT was moderately sensitive for detecting unilateral and bilateral vestibulopathy overall". A negative aspect of this study is that the functional tests were not performed for the superior (which innervates the anterior and horizontal semicircular canal and the utricle) and the inferior (which innervates the posterior semicircular canal and the saccule) vestibular nerve separately. In future, it would be better to distinguish the pathology in the vestibular organ using different functional vestibular tests: to perform caloric testing and vHIT for all semicircular canals, as there seems to be a higher sensitivity in caloric testing than vHIT [18], but it is advisable to perform vHIT first, as there is a lower burden for the patient in acute vestibular neuropathy than if performing caloric testing [19]. Additionally, cervical vestibular evoked myogenic potentials (cVEMP) to test the function of the saccule and ocular vestibular myogenic potentials (oVEMP) to test the utricle can be performed [20-21]. Lee et al. [20] distinguish between three different versions of vestibular neuritis depending on the site of involvement: total VN is defined as VN with involvement of the superior and inferior vestibular nerve; superior VN is defined by involvement of the superior vestibular nerve; and inferior VN by the involvement of the inferior vestibular nerve. For the detection of these differences, the authors performed videonystagmography (VNG), vHIT, caloric testing, and cVEMP. It is advised to perform VEMP in suspected cases of superior canal dehiscence syndrome [19]. Another additional functional test is the bucket test, which is a tool to determine the subjective visual vertical [8].

Laboratory findings

In this study, the mean leukocyte count was 9717 ± 2991 /μl and leukocytes were elevated in 39 patients (39.8%) with a mean value of 12 344 ± 1703 /μl and CRP was elevated in eight patients (8.2%) with a mean value of 21.9 ± 10.1 mg/dl. Kassner et al. [13] also found elevated leukocytes (8.17 ± 0.87) in their VN patients compared to their control group with no statistically significant difference and a 2.2-fold elevated CRP level. In their study, Chung et al. [14] asserted that the numbers of white blood cells (8.76 ± 2.84), lymphocytes and monocytes of VN patients were elevated, but not statistically significant compared to the control group, whereas their NLR and PLR values were significantly higher in the VN patient group (NLR=3.31, PLR=129.5) compared to the healthy control group (NLR=1.60, PLR=103.44). They also found a positive correlation between the duration of vertigo and NLR and PLR. In our study, NLR (3.63 ± 3.21) and PLR (147.75 ± 67.47) were even higher than in the study by Chung et al. [14]. These results confirm the hypothesis of earlier studies, which states that VN patients have elevated leukocytes and CRP in many cases.

In a recently published study by Rujescu et al. [22], an association was found between four genetic regions in VN patients with a link to viral infections such as HSV-1 and HIV-1 and a link to insulin metabolism (and therefore to chronic infection). These findings support the theory of viral infection in VN patients. The theory of reactivation of latent infections with viruses such as HSV-1 and HSV-6 [17-20] was also confirmed in studies by Arbusow et al. [23] and Furuta et al. [24], where HSV DNA was found in human vestibular ganglia in autopsied temporal bones. In our study, HSV IgG was elevated in 52.8% of patients tested; furthermore, there were four patients (7.5% of the serologically tested VN patients) who were positive for HSV IgM. The prevalence of CMV in industrial countries is about 40-70% [25]. CMV IgG was elevated in 24 patients (45.3%) and CMV IgM in one patient (1.9%). There was positive IgG for EBV in 19 patients (35.8%), which is low compared to the prevalence in industrial nations, which is up to 95% by age 30. Six patients (11.3%) tested positive for LD IgM, three were positive for IgG (5.7%) and two of them (3.8%) were also LD positive when using the immunoblot confirmation test. Walther et al. [26] reported a prevalence of Lyme disease of 18%, whereas Hydén et al. [27] reported a 2% prevalence. Treatment of VN patients who test positive for LD or HSV might be optimized with oral antibiotics or antiviral therapy according to the guidelines. There are only a few studies that deal with the relationship between LD and VN. Further research is needed to clarify if LD and other neurotropic infections are one of the causes of VN. Since the COVID-19 pandemic, there are also reports about VN after COVID-19 infection and after vaccination [28] and the reports about its neurotropic potential accumulate. Therefore it is essential to test patients with acute onset of peripheral vestibular neuropathy also for COVID-19.

Imaging findings

Imaging is another tool, which can be used to confirm the diagnosis of vestibular neuritis. As MR imaging has become more detailed and specific in certain situations and the availability has increased, it seems to be possible to visualize the enhanced vestibular nerve in VN patients. Therefore, usually, a gadolinium-enhanced T1-weighted MRI is performed. In one out of 30 patients (3.3%) in this study, the images revealed an enhancement of the vestibular nerve in the delayed post-contrast MRI sequences (Figure 2). The low number of enhancing vestibular nerves in MRI in this study might be due to nonspecific MRIimaging for this question. MRI imaging was performed to rule out the possibility of a neurologic, central pathology. Byun et al. [29] reported on four-hour delayed gadolinium-enhanced 3D fluid-attenuated inversion recovery (FLAIR) MR images in VN patients and were able to visualize the enhanced nerve in 69% of their patients. Park et al. [30] reported the possibility to use three-dimensional FLAIR-VISTA MRI to detect nerve enhancement.

Limitations and perspectives

The strength of this study is that it includes a large number of cases and the evaluation of various clinical parameters. Limitations of the study are a retrospective study design and therefore missing data in some cases in regard to laboratory parameters and functional diagnostics. It is advisable to perform caloric testing, VNG and vHIT for all semicircular canals and additionally cervical vestibular evoked myogenic potentials (cVEMP) to test the function of the saccule and ocular vestibular myogenic potentials (oVEMP) to test the utricle. Additional diagnostics with the rotary chair may be helpful and add information in cases of suspected bilateral VN. Another interesting aspect is nerve enhancement in MR imaging and if available, it may confirm the diagnosis of VN. 

A prospective multi-center study with the inclusion of the above-mentioned functional diagnostics and laboratory testing for various neurotropic infectious diseases, especially HSV, LD and COVID-19, could help to gather more information about prevalences and the possibilities of a specific treatment, for example with antiviral or antimicrobial therapy.

## Conclusions

Patients with VN had a high proportion of pathologically elevated infection parameters. In addition to the detection of acute specific infections such as LD and HSV infection, a non-specific increase in leukocytes and high NLR and PLR were observed in the majority of cases. A dependence on concomitant diseases could not be demonstrated. Due to a high prevalence of LD in this study we recommend diagnostic workup in suspected VN testing not only for HSV but also for LD and COVID-19. This approach enables complementing the standard VN treatment with a causal treatment. In cases of infectious VN, using the term vestibular neuritis is justified.
